# Which Is Better for Liver SBRT: Dosimetric Comparison Between DCAT and VMAT for Liver Tumors

**DOI:** 10.3389/fonc.2020.01170

**Published:** 2020-07-29

**Authors:** Young Min Moon, Wan Jeon, Tosol Yu, Sang Il Bae, Jin Young Kim, Jin-Kyu Kang, Chul Won Choi

**Affiliations:** Department of Radiation Oncology, Dongnam Institute of Radiological and Medical Science, Busan, South Korea

**Keywords:** liver cancer, SBRT (stereotactic body radiotherapy), VMAT (volumetric modulated arc therapy), Monaco TPS (treatment planning system), DCAT (dynamic conformal arc therapy)

## Abstract

Stereotactic body radiotherapy (SBRT) is currently well-adopted as a curative treatment for primary and metastatic liver tumors. Among SBRT methods, dynamic conformal arc therapy (DCAT) and volumetric-modulated arc therapy (VMAT) are the most preferred methods. In this study, we report a comparison study measuring the dose distribution and delivery efficiency differences between DCAT and VMAT for liver SBRT. All patients who were treated with SBRT for primary or metastatic liver tumors with a curative aim between January 2016 and December 2017 at DIRAMS were enrolled in the study. For all patients, SBRT plans were designed using the Monte Carlo (MC) algorithm in Monaco treatment planning system (version 5.1). The planning goals were set according to the RTOG 0813, RTOG 0915, and RTOG 1112 protocols. A plan comparison was made on the metrics of dose volume histogram, planning and delivery efficiency, monitor unit (MU), and dosimetric indices. PTV coverage was evaluated using the following: D_mean_, D95%, D98%, D2%, D50%, D_max_, V95%, heterogeneity index (HI), and conformality index (CI). For DCAT and VMAT, respectively, the D_mean_ was 5942.8 ± 409.3 cGy and 5890.6 ± 438.8 cGy, D50% was 5968.8 ± 413.1 cGy and 5954.3 ± 405.2 cGy, and CI was 1.05 ± 0.05 and 1.03 ± 0.04. The D98% and V95% were 5580.0 ± 465.3 cGy and 20.4 ± 12.0 mL for DCAT, and 5596.0 ± 478.7 cGy and 20.5 ± 12.0 mL for VMAT, respectively. For normal liver, V40, V30, V20, V17, V5, D_mean_, D_max_ were evaluated for comparison. The V30, V20, and V10 were significantly higher in DCAT; other parameters of normal livers showed no statistically significant differences. For evaluation of intermediate dose spillage, D2_cm_(%) and R50% of DCAT and VMAT were 45.8 ± 7.9 and 5.6 ± 0.9 and 45.1 ± 6.7 and 5.5 ± 1.2, respectively. Planning and delivery efficiency were evaluated using MU, Calculation time, and Delivery time. DCAT had shorter Calculation time and Delivery time with smaller MU. MU was smaller in DCAT and the average difference was 300.1 MU. For liver SBRT, DCAT is an effective alternative to VMAT plans that could meet the planning goals proposed by the RTOG SBRT protocol and increases plan and delivery effectiveness, while also ignoring the interplay effect.

## Introduction

Stereotactic body radiotherapy (SBRT) is currently well-adopted as a curative treatment for primary and metastatic liver tumors, especially for patients who are inoperable or undergoing systemic therapy (1–4). SBRT can be performed by various methods, including 3D conformal radiotherapy (3DCRT), intensity modulated radiotherapy (IMRT), dynamic conformal arc therapy (DCAT), volumetric-modulated arc therapy (VMAT), Tomotherapy, and CyberKnife. However, VMAT and DCAT are the most preferred methods (5–7). Both VMAT and DCAT are types of rotational radiotherapy, with each system having its own advantages and disadvantages. VMAT is typically considered superior to DCAT in terms of dose distribution since it has better target coverage. However, DCAT has potential advantages over VMAT in practical use. Reasons to consider DCAT instead of VMAT (8, 11) are as follows: (1) There is a concern about missing the target when VMAT is used for a moving target. Even if DCAT does not cover all targets of irregular movement, at least DCAT might offset this concern for the effect of MLC interplay since the target remains inside the open field with minimal modulation for the entire treatment. (2) VMAT requires a higher degree of quality assurance. (3) There is less concern with calculation accuracy for DCAT in an area of variable densities. (4) DCAT offers quicker plan and delivery times. (5) DCAT demands less monitor unit (MU) to deliver the same dose as VMAT. Furthermore, segment shape optimization (SSO) could supplement DCAT to maintain its inherent advantages while achieving results similar to VMAT. SSO is offered by the Monaco treatment planning system (TPS) (IMPAC Medical Systems, Inc., Maryland Heights, MO; a subsidiary of Elekta AB, Stockholm, Sweden) to improve plan quality by smoothing and clustering segments and optimizing beam weights and shapes. If DCAT for liver tumors is not inferior with regard to dose distribution and satisfies RTOG guidelines, DCAT could be a better option for SBRT plans than VMAT. Here, we report a comparison study measuring the dose distribution and delivery efficiency differences between DCAT and VMAT. Suggestions are offered for improved plan technique in primary and metastatic liver tumor radiotherapy.

## Materials and Methods

This study was approved by the institutional review board of the Dongnam Institute of Radiological and Medical Sciences (DIRAMS).

### Patient Selection

All patients who were treated with SBRT for primary or metastatic liver tumors with a curative aim between January 2016 and December 2017 at DIRAMS were enrolled in the study. Patients with palliative aims, such as SBRT for portal vein tumor thrombosis, were excluded. All plans were built based on the prescribed dose at that time of treatment.

### Contouring

The target volumes and organs at risk (OARs) of all included patients were reviewed and re-contoured by one radiation oncologist. Gross target volume (GTV) was determined by merging the re-drawn tumor volumes for each phase of the four-dimensional computed tomography (4DCT). Clinical target volume was same as GTV, and plan target volume (PTV) was established with 3–8 mm expansion of the GTV. The total volume of the liver, heart, stomach, and duodenum were contoured. Other OARs were partially drawn as needed for treatment planning.

### Treatment Planning

**Figure 1:**
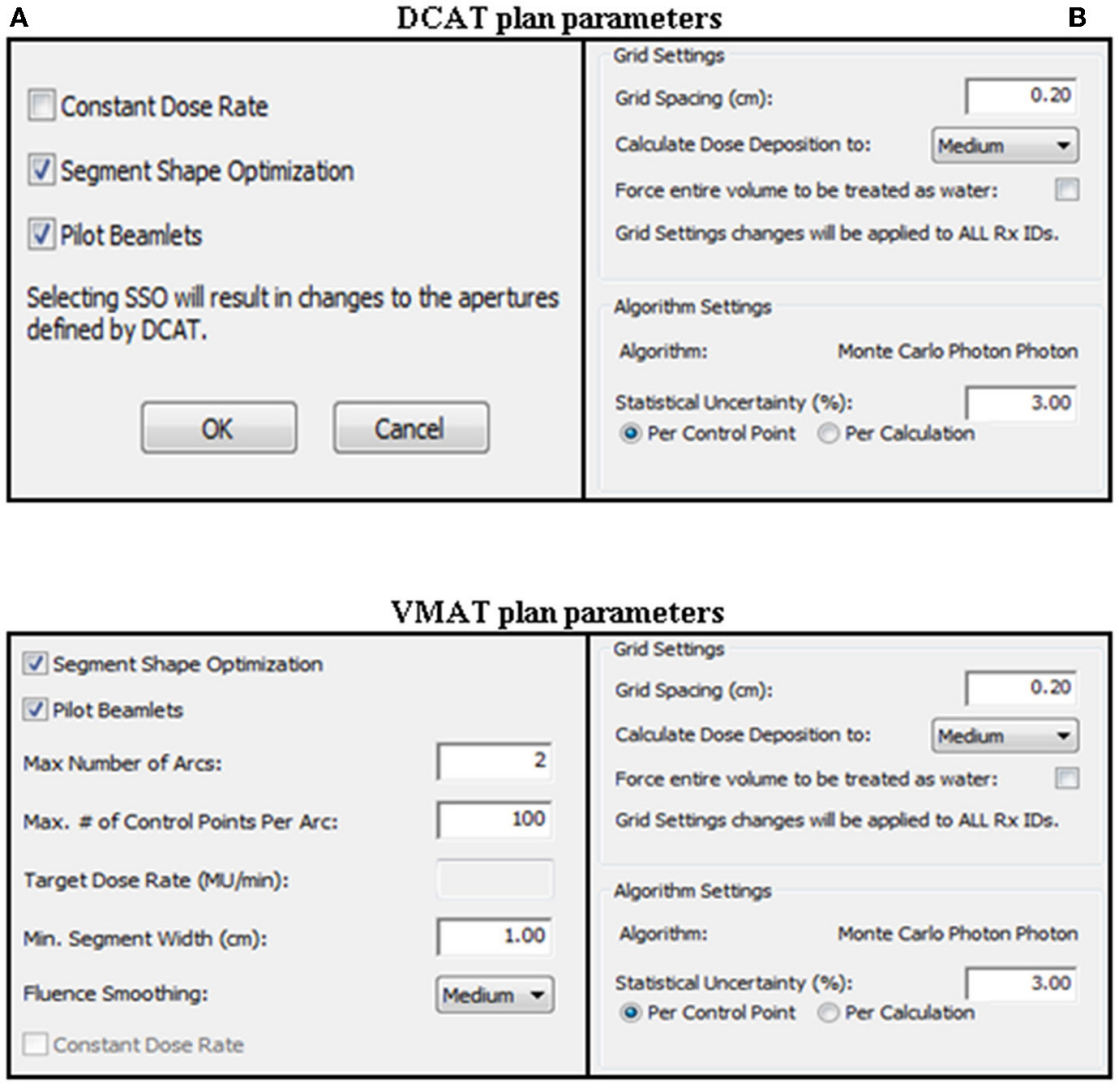
Physical parameters for DCAT and VMAT plans. **(A)** Sequencing parameters. **(B)** Calculation parameters.

For all patients, SBRT plans were designed using the MC algorithm in Monaco TPS (version 5.1). Beam modeling of the MONACO TPS was performed by beam of Elekta Infinity linear accelerator (Elekta AB, Stockholm, Sweden). All plans were designed using a photon beam of energy 6 MV with nominal dose rate 600 MU/min. To accompany the prescription and dose criteria, constraints of the plan optimization were adjusted depending on the size and location of the tumor. However, the plans were developed with the same constraints to allow comparison of the DCAT plan with the VMAT plan for each patient. After planning optimization, all plans were normalized to 95% of the PTV coverage with the prescribed dose. The physical parameters for each plan are shown in Figure 1. Each application of DCAT was planned as a single arc with non-constant dose rate and SSO applied. The VMAT plan was designed using a dual arc with a maximum 150 control points per arc and 1.0 cm minimum segment width. The arc length of both plan types was the same, with a range of 360° rotating from 180° in increments of 10° without couch rotation using fluence-smoothing parameters in medium mode. The final dose calculation was performed using a calculation grid resolution of 2.0 mm and a statistical uncertainty of 3% per control point. Physical parameters such as calculation grid resolution, statistical uncertainty, arc length, number of arcs, increment, number of control point/arc, and fluence-smoothing parameters were identical for each case in the same type of plan. The planning goals were set according to the RTOG 0813, RTOG 0915, and RTOG 1112 protocols.

### Statistics

Statistical analysis was performed using the SPSS 18.0 program. Data normality was assessed using the Shapiro-Wilk test. Student's paired *t*-test and the Wilcoxon signed-rank test were used, respectively, for parametric and non-parametric data analyses (9).

## Results

### Patient Characteristics

Twenty-five patients with a total of 31 lesions were enrolled. Two patients had 3 lesions, and 2 patients had 2 lesions, simultaneously. In one patient with 3 lesions, 2 of the lesions were located close to each other and were combined into a single PTV. Among the 25 patients, 17 had hepatocellular carcinomas (HCCs), 8 had metastatic liver cancers, and 7 had received previous radiotherapy (RT). A total of 30 plans for each arm were made and evaluated for comparison. The prescribed SBRT dose was 4,800–6,000 cGy in 4 or 5 fractions. The characteristics of the patients are summarized in Table 1.

**Table 1:** Patient characteristics.

**Patient no**.	**Lesions**	**Primary cancer**	**Previous RT to Liver**	**Location**	**GTV (mL)**	**PTV (mL)**	**Prescribed dose (cGy)**	**Fractions**
1	1	HCC	–	S4	5.1	20.1	5,200	4
2	1	HCC	–	S7	1.0	14.0	6,000	4
3	1	HCC	+	S7	5.4	19.2	5,400	4
4	1	HCC	+	S8	4.2	16.0	5,000	5
5	1	Cervix	–	S6	1.9	10.8	4,800	4
6	1	HCC	–	S5	7.7	15.9	6,000	4
7	1	HCC	–	S7/8	7.1	24.5	6,000	4
8	1	HCC	–	S7/8	10.6	31.4	6,000	4
9	1	HCC	–	S7	22.1	53.5	6,000	4
10	1	HCC	–	S7	8.7	31.9	6,000	4
11	3	Pancreas	–	S8, S4, S6	3.5, 6.9, 3.1	23.3, 23.2, 14.1	5,400	4
12	3	HCC	–	LLS, S4/8	1.1, 1.2, 2.1	24.3, 19.4	5,400	4
13	1	HCC	+	S8	7.9	14.3	6,000	4
14	1	HCC	–	S6	6.2	22.7	6,000	4
15	1	HCC	–	S8	11.5	37.4	6,000	4
16	1	HCC	–	S7	1.6	11.6	6,000	4
17	1	HCC	+	S4	3.7	15.5	6,000	4
18	1	Rectum	–	S7	0.4	6.4	6,000	4
19	1	Cervix	–	S4	7.6	22.9	6,000	5
20	1	Rectosigmoid	+	S4/8	24.0	58.7	5,000	4
21	2	Rectosigmoid	–	S8, S6	2.0, 1.2	10.9, 7.7	6,000	4
22	2	Colon	+	S5, S5	1.6, 1.7	11.4, 11.5	6,000	4
23	1	HCC	–	S5	4.0	11.5	6,000	4
24	1	HCC	–	S4	4.2	15.7	5,000	5
25	1	Rectosigmoid	+	S8	3.0	14.9	6,000	4

*No., number; RT, radiotherapy; GTV, gross target volume; PTV, planning target volume*.

### Dose Distribution

**Figure 2:**
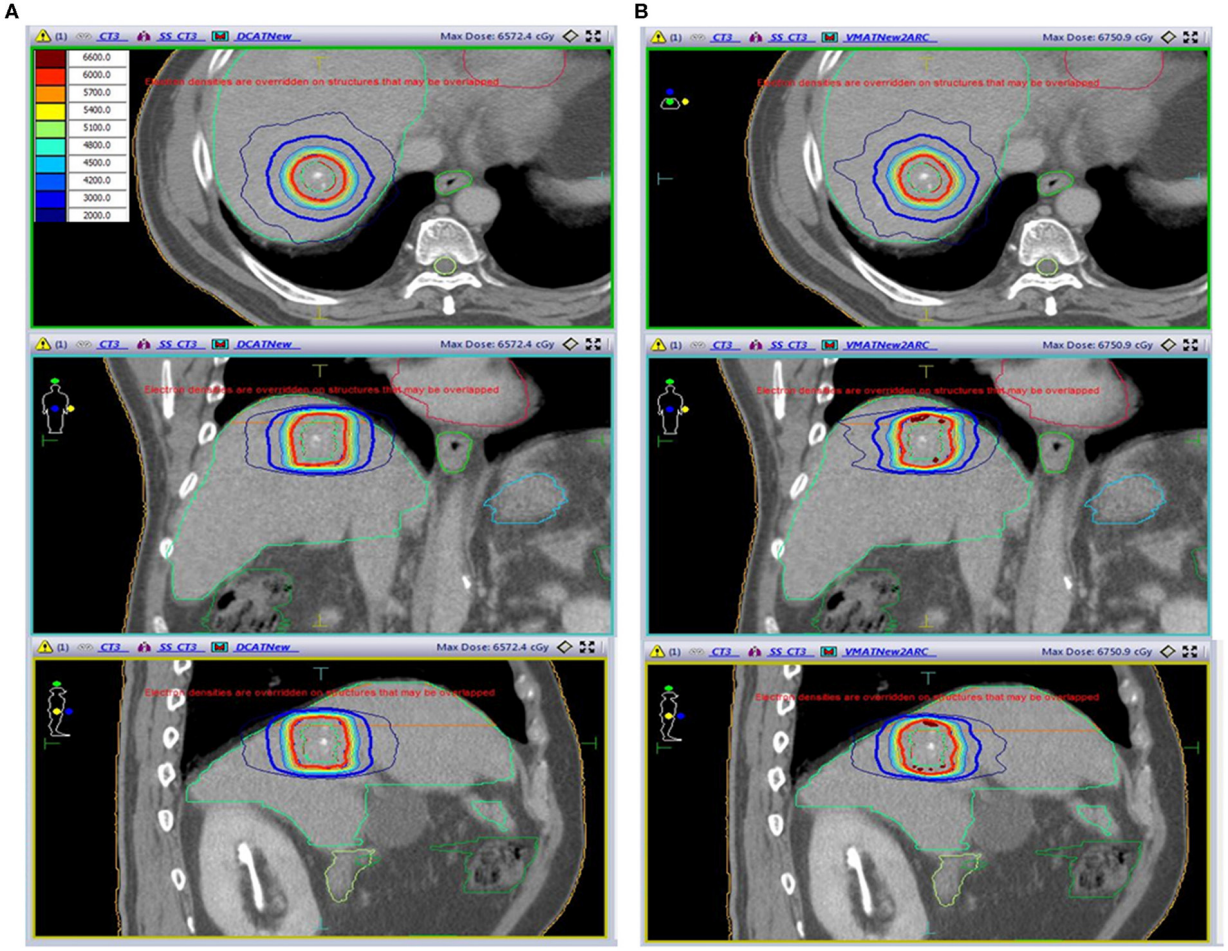
The dose distribution for the same patient was shown in axial, coronal and sagittal planes according to the plan type. **(A)** DCAT. **(B)** VMAT.

**Figure 3:**
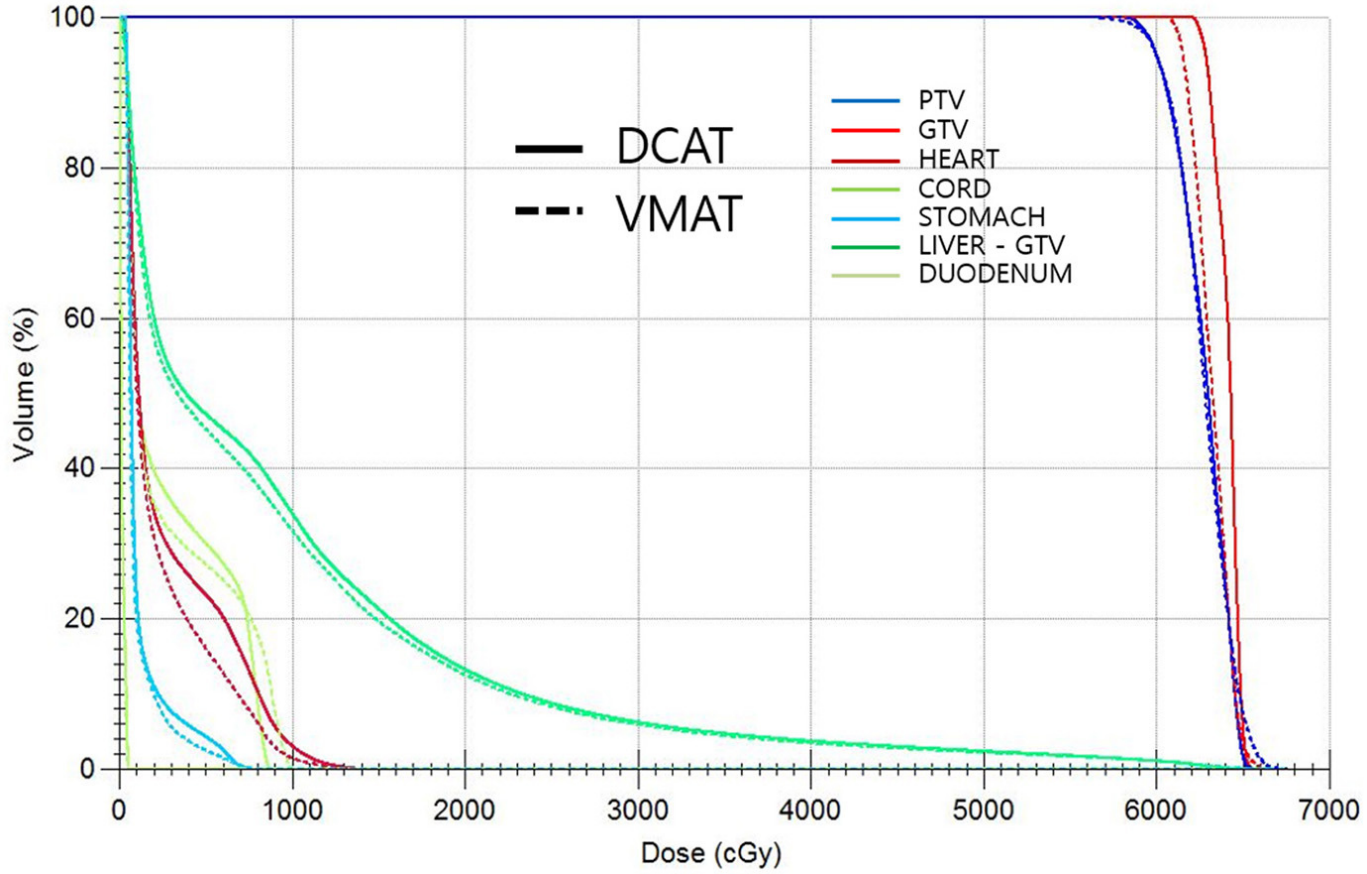
Comparison of DVHs between DCAT and VMAT plan for the same patient.

All DCAT and VMAT plans were optimized with an identical optimization protocol, and all except 4 patient plans achieved the planning goals. According to plan type, the dose distribution and DVH for the same patient are shown in Figures 2, 3, respectively. Four patients' plans could not reach the objectives due to close proximity to OARs and some goals were compromised according to the physician's discretion to comply with the dose constraints.

PTV coverage was evaluated using the following: mean dose (D_mean_), D95%, D98% (near-minimum absorbed dose), D2% (near-maximum absorbed dose), D50%, maximum point dose to 0.035 mL (D_max_), V95%, heterogeneity index (HI), and conformality index (CI). Among the evaluated parameters, D_mean_, D98%, D50%, V95%, and CI showed statistically significant differences between DCAT and VMAT. The D_mean_, D50%, and CI were higher, and D98% and V95% were lower in DCAT than in VMAT. For DCAT and VMAT, respectively, the D_mean_ was 5942.8 ± 409.3 cGy and 5890.6 ± 438.8 cGy, D50% was 5968.8 ± 413.1 cGy and 5954.3 ± 405.2 cGy, and CI was 1.05 ± 0.05 and 1.03 ± 0.04. The D98% and V95% were 5580.0 ± 465.3 cGy and 20.4 ± 12.0 mL for DCAT, and 5596.0 ± 478.7 cGy and 20.5 ± 12.0 mL for VMAT, respectively. The data for PTV dose distribution are presented in Table 2.

**Table 2:** PTV coverage.

	**DCAT**	**VAMT**	**Avg. difference (DCAT – VMAT)**	***p*–value**
D_max_ (cGy)	6199.1 ± 419.1	6177.7 ± 428.4	21.4 ± 95.9	0.231
D_mean_ (cGy)	5942.7 ± 409.3	5890.6 ± 438.8	52.2 ± 194.3	0.015
D95% (cGy)	5678.4 ± 425.2	5680.9 ± 421.3	−2.5 ± 12.6	0.050
D98% (cGy)	5580.0 ± 465.2	5596.0 ± 478.7	−16.1 ± 44.9	0.005
D2% (cGy)	5981.1 ± 1100.8	6126.0 ± 434.2	−145.0 ± 960.2	0.289
D50% (cGy)	5968.8 ± 413.1	5954.3 ± 405.2	14.5 ± 73.6	0.042
V95% (mL)	23.5 ± 18.6	23.5 ± 18.6	−0.1 ± 0.7	0.022
HI	1.08 ± 0.04	1.07 ± 0.04	0.01 ± 0.02	0.062
CI	1.05 ± 0.05	1.03 ± 0.04	0.02 ± 0.03	0.003

*Avg., average; CI, conformality index; HI, heterogeneity index; PTV, planning target volume*.

For normal livers (liver volume minus GTV), V40, V30, V20, V17, V5, D_mean_, D_max_ were evaluated for comparison. The V30, V20, and V10 were significantly higher in DCAT; other parameters of normal livers showed no statistically significant differences. D_max_, maximum dose to 1 mL (D_1mL_), maximum dose to 2 mL (D_2mL_), and D_mean_ were assessed for OARs such as the duodenum, stomach, small bowel, large bowel, spinal cord, heart, and esophagus. For the duodenum, all assessed parameters show statistically significant differences. D_max_, D_1mL_, and D_2mL_ were higher in DCAT, but the D_mean_ was lower than in VMAT. For both the stomach and heart, D_max_, D_1mL_, D_2mL_ were significantly higher in DCAT. The dose data for OARs that show statistically significant differences are listed in Table 3 and entire data in Supplementary Table 1.

**Table 3:** Statistically significant differences in OARs.

	**DCAT**	**VMAT**	**Avg. difference (DCAT – VMAT)**
V30 (mL)	65.9 ± 34.4	62.1 ± 29.8	3.8 ± 9.2
V20 (mL)	133.7 ± 65.4	125.6 ± 65.4	8.1 ± 17.2
V10 (mL)	346.2 ± 196.7	318.9 ± 165.3	27.3 ± 48.4
Duodenum D_max_ (cGy)	627.6 ± 1065.3	615.82 ± 1046.7	11.8 ± 119.1
Duodenum D_1mL_ (cGy)	478.9 ± 757.1	461.7 ± 724.1	17.2 ± 123.6
Duodenum D_2mL_ (cGy)	395.3 ± 620.4	378.5 ± 581.6	16.8 ± 136.1
Duodenum D_mean_ (cGy)	85.6 ± 118.8	88.2 ± 130.3	−2.6 ± 56.7
Stomach D_max_ (cGy)	1057.2 ± 1042.6	968.0 ± 1046.4	89.1 ± 191.3
Stomach D_1mL_ (cGy)	913.3 ± 829.5	826.6 ± 796.0	86.7 ± 178.4
Stomach D_2mL_ (cGy)	855.9 ± 755.8	775.0 ± 708.4	80.9 ± 176.9
Heart D_max_ (cGy)	1439.7 ± 1589.5	1361.8 ± 1553.6	77.9 ± 185.2
Heart D_1mL_ (cGy)	1250.4 ± 1354.3	1175.3 ± 1326.4	75.1 ± 176.8
Heart D_2mL_ (cGy)	1167.8 ± 1233.6	1095.0 ± 1214.6	72.8 ± 174.0

*Avg., average; D_mean_, mean dose; D_max_, maximum dose*.

The maximum dose to any point ≥2 cm away from the PTV in any direction (D_2cm_) and the ratio of the volume of 50% of the prescription dose isodose to the volume of the PTV (R_50%_) are reported for evaluation of intermediate dose spillage and to scrutinize the fall-off gradient beyond the PTV extending into normal tissue structures. The D_2cm_(%) and R_50%_ of DCAT and VMAT were 45.8 ± 7.9 and 5.6 ± 0.9, and 45.1 ± 6.7 and 5.5 ± 1.2, respectively. The differences were not statistically significant. The D_2cm_(%) and R_50%_ data for all patients are shown in Table 4.

**Table 4:** D_2cm(%)_ and R_50%_ for all cases.

**Case No**.	**PTV (mL)**	**D**_****2cm(%)****_		**R**_****50%****_	
		**DCAT**	**VMAT**	**DCAT**	**VMAT**
1	20.1	43.3	45.9	4.7	4.3
2	14.0	41.8	43.2	5.3	4.9
3	19.2	42.3	42.6	4.5	4.6
4	16.0	42.2	44.5	5.8	5.6
5	10.8	42.5	42.3	5.8	5.3
6	15.9	43.1	42.5	5.7	5.0
7	24.5	43.1	43.9	4.6	4.4
8	31.4	45.9	43.4	5.1	4.2
9	53.5	49.1	48.1	4.3	4.0
10	31.9	44.8	46.5	4.5	4.5
11	23.3	43.8	44.3	5.3	4.9
12	23.2	41.8	42.0	4.8	5.1
13	14.1	50.5	49.7	5.8	6.2
14	24.3.	51.0	51.2	5.6	6.0
15	19.4	46.2	42.7	5.6	4.5
16	14.3	41.0	41.6	5.9	6.0
17	22.7	42.6	43.2	5.0	4.8
18	37.4	44.3	45.0	4.2	4.1
19	11.6	41.4	41.3	6.2	6.4
20	15.5	41.6	41.5	5.6	5.7
21	6.4	40.6	40.0	8.0	8.8
22	22.9	83.8	76.7	7.4	6.0
23	58.7	52.1	50.0	5.5	4.7
24	10.9	48.1	45.8	6.8	7.2
25	7.7	47.6	47.3	7.7	8.1
26	11.4	42.5	39.7	5.8	6.2
27	11.5	42.0	40.2	5.7	6.5
28	15.7	44.4	42.4	5.5	5.4
29	14.9	47.4	44.7	5.8	6.0
30	13.9	41.9	41.7	5.9	6.1

*No., number; PTV, planning target volume*.

### Efficiency

We also evaluated plan and delivery efficiency using MU, the elapsed time to make a plan (Calculation time), and the estimated delivery time (Delivery time). The MU of DCAT and VMAT were 2440.5 ± 346.5 and 2741.4 ± 417.5, respectively, and the average difference was 300.9 ± 340.4. Calculation time for DCAT and VMAT was 14.4 ± 7.5 min and 29.0 ± 14.2 min, and delivery time was 3.6 ± 0.5 min and 4.5 ± 0.7 min, respectively. All efficiency parameters analyzed showed statistically significant differences (Table 5).

**Table 5:** Calculation time, Delivery time, and MU.

	**DCAT**	**VMAT**	**Avg. difference (DCAT – VMAT)**
Calculation time (min)	14.4 ± 7.5	29.0 ± 14.2	−14.6 ± 15.2
Delivery time (min)	3.6 ± 0.5	4.5 ± 0.7	−0.9 ± 0.6
MU	2440.5 ± 340.4	2741.4 ± 417.5	−300.9 ± 340.4

*Delivery time and MU for 1 fraction of stereotactic body radiotherapy*.

*Avg., average; MU, monitor unit*.

## Discussion

For plan comparison, we used 1 arc for DCAT and 2 arcs for VMAT. One-arc and 2-arc plans with both DCAT and VMAT were used for some patients to verify the ideal number of arcs for each method. Dose distribution in DCAT showed little improvement from increment of arc numbers. In contrast, VMAT showed better results with 2 arcs than with 1 arc. For comparison with the best plans, 1 arc in DCAT and 2 arcs in VMAT were used.

In this study, couch rotation was not used due to disadvantages of couch rotation. Even though the use of couch rotation can help improve dosimetric parameters, couch rotation should be used with caution because the theoretical advantage could be offset by errors caused by an increase in treatment time and position changes.

As expected, VMAT was superior to DCAT to build SBRT plan with better plan quality. However, although there were some statistically significant differences between DCAT and VMAT plans, we could not assert if VMAT was better than DCAT with only dose distribution in the absence of consideration for actual use. As mentioned above, DCAT has several advantages over VMAT that could offset the better dose-distribution of VMAT. Unless the differences of dose distribution and plan quality are substantial, DCAT could be reckoned prior to VMAT for SBRT in some specific cases. There are concerns regarding missing the targets in highly modulated treatment plan for moving targets such as liver tumors. Liver tumors might not only move up and down but also move sideways and twist. Moreover, liver tumors tend to grow in a relatively circular shape and this suggests that DCAT would be as appropriate as VMAT to achieve adequate target coverage in liver tumors. That is the one of reason why we should consider DCAT for liver tumors during SBRT if the differences between DCAT and VMAT are comparable. In addition, complex interplay between MLC motion, jaw movement, gantry rotation, and target motion during free-breathing treatments with VMAT could cause considerable dose discrepancies (7, 10). Our results indicated that several dosimetric parameters were significantly better in VMAT plans. However, DCAT also met the plan goals proposed by the RTOG SBRT protocol and the dose distribution differences were small enough to accept DCAT as a method of SBRT instead of VMAT.

D_2cm_(%) and R_50%_ values are originally proposed for lung SBRT but not for the liver. However, we wanted to manage and minimize spillage dose and hence, D_2cm(%)_ and R_50%_ were evaluated. D_2cm(%)_ achieved the suggested goal except in 1 case. In this 1 case where the D_2cm(%)_ was not achieved, the tumor was located near the duodenum and D_2cm(%)_ was compromised to obtain acceptable dose distribution to the duodenum. Meanwhile, R_50%_ failed to meet the goal in 12 cases. This might be due to the different prescription goals for liver and lung PTV in RTOG protocols. To clarify the reason why R_50%_ is hard to achieve, we tested dose spillage through CT scans with lung and liver density and there was no significant difference according to tissue densities. Stathakis et al. recently published the study of dosimetric comparison between VMAT and DCAT in SBRT for the lung and liver (10). In that study, R_50%_ seemed to reach the desired goal; however, there appeared to be calculation errors in their R50 values. Though differences in protocols make it difficult to achieve R50 in the liver, there is still a need to evaluate controlling dose spillage outside the PTV.

In terms of efficiency, DCAT had shorter calculation times and delivery times with smaller MU. Since calculation time changes variably depending on the number of programs running on the TPS at the same time, measurement of accurate time for planning is not available. Nevertheless, calculation time is generally 2–3 times longer in VMAT, and DCAT could allow for quicker plans. Shorter delivery times would help patients maintain treatment postures and to control breathing which would reduce target misses during RT. Therefore, DCAT should be considered preferential to VMAT in patients with poor breathing or poor coordination. MU was smaller in DCAT; the average difference was 300.1 MU. The use of smaller MU would reduce both the load on the LINAC machine and concerns about leakage through the multi-leaf collimator. In addition, DCAT could help conserve resources, which might be important to institutions with many patients but limited resources.

Before SBRT, some patients receive prior surgery or RT and would need stricter dose control to avoid severe toxicities. VMAT would be more appropriate in such patients for minimizing the dose to OARs, although the dose differences are small. Moreover, since DCAT is not recommended for constructing plans for multiple lesions, VMAT should be considered when SBRT is administered for multiple lesions simultaneously. However, if multiple lesions are treated separately, DCAT should be given the priority.

As noted above, DCAT might be more advantageous than VMAT for liver SBRT with exception to specific cases. Although the method of treatment is at the physician's discretion, the physician needs to understand the pros and cons of each treatment prior to choosing a treatment method. We hope this study will help radiation oncologists make better decisions for selection of SBRT methods.

## Data Availability Statement

All datasets generated for this study are included in the article/Supplementary Material.

## Ethics Statement

This study was retrospective and exempted from obtaining written informed consent. The study was also approved by the Institutional Review Board of the Dongnam Institute of Radiological and Medical Sciences (DIRAMS).

## Author Contributions

All authors listed have made a substantial, direct and intellectual contribution to the work, and approved it for publication.

## Conflict of Interest

The authors declare that the research was conducted in the absence of any commercial or financial relationships that could be construed as a potential conflict of interest.
