# Supplementary material for: Which Is Better for Liver SBRT: Dosimetric Comparison Between DCAT and VMAT for Liver Tumors
Source: Front Oncol. 2020 Jul 29;10:1170. doi: 10.3389/fonc.2020.01170 (PMC7403186; doi:10.3389/fonc.2020.01170)
Supplement: Supplementary file 1 [file Table_1.DOCX]

Supplementary Table 1. Entire dose distribution data of OARs

|  | DCAT (cGy) | VMAT (cGy) | *p*-value |
| --- | --- | --- | --- |
| Liver |  |  |  |
| V40 | 40.4 ± 20.3 | 38..4 ± 34.4 | 0.054 |
| V30 | 65.9 ± 34.4 | 62.1 ± 29.8 | 0.040 |
| V20 | 133.7 ±74.6 | 125.6 ± 65.4 | 0.018 |
| V17 | 173.6 ± 100.2 | 164.5 ± 87.9 | 0.094 |
| V10 | 346.2 ± 196.7 | 318.9 ± 165.3 | 0.003 |
| V5 | 515.1 ± 273.0 | 505.1 ± 262.8 | 0.319 |
| D_mean_ | 718.2 ± 310.4 | 692.5 ± 288.4 | 0.066 |
| D_max_ | 6144.3 ± 413.9 | 6139.3 ± 440.6 | 0.796 |
| Duodenum |  |  |  |
| D_max_ | 627.6 ± 1065.3 | 615.8 ± 1046.7 | 0.017 |
| D_1mL_ | 478.9 ± 757.1 | 461.7 ± 724.1 | 0.009 |
| D_2mL_ | 395.3 ± 620.4 | 378.5 ± 581.6 | 0.010 |
| D_mean_ | 85.6 ± 118.8 | 88.2 ± 130.3 | 0.013 |
| Stomach |  |  |  |
| D_max_ | 1057.2 ± 1042.6 | 968.0 ± 1046.4 | 0.016 |
| D_1mL_ | 913.3 ± 829.5 | 826.6 ± 796.0 | 0.020 |
| D_2mL_ | 855.9 ± 755.8 | 775.0 ± 708.4 | 0.017 |
| D_mean_ | 194.3 ± 166.4 | 180.5 ± 153.8 | 0.217 |
| Small bowel |  |  |  |
| D_max_ | 429.3 ± 1478.1 | 199.4 ± 254.7 | 0.603 |
| D_1mL_ | 153.2 ± 197.6 | 175.0 ± 230.0 | 0.866 |
| D_2mL_ | 142.7 ± 182.3 | 162.4 ± 217.2 | 0.839 |
| D_mean_ | 46.6 ± 53.6 | 50.3 ± 70.0 | 0.732 |
| Large bowel |  |  |  |
| D_max_ | 777.5 ± 884.6 | 747.9 ± 822.2 | 0.665 |
| D_1mL_ | 666.3 ± 727.5 | 629.0 ± 654.7 | 0.540 |
| D_2mL_ | 616.9 ± 669.1 | 577.4 ± 594.2 | 0.509 |
| D_mean_ | 80.6 ± 126.5 | 77.8 ± 100.0 | 0.288 |
| Spinal cord |  |  |  |
| D_max_ | 829.0 ± 256.1 | 834.6 ± 304.2 | 0.894 |
| D_1mL_ | 752.7 ± 237.4 | 756.5 ± 279.8 | 0.991 |
| D_2mL_ | 695.2 ± 230.7 | 697.9 ± 275.1 | 0.992 |
| D_mean_ | 217.3 ± 109.2 | 218.7 ± 112.5 | 0.815 |
| Heart |  |  |  |
| D_max_ | 1439.7 ± 1589.5 | 1361.8 ± 1553.6 | 0.041 |
| D_1mL_ | 1250.4 ± 1354.3 | 1175.3 ± 1326.4 | 0.047 |
| D_2mL_ | 1167.8 ± 1233.6 | 1095.0 ± 1214.6 | 0.047 |
| D_mean_ | 239.7 ± 237.9 | 218.6 ± 219.1 | 0.096 |
| Esophagus |  |  |  |
| D_max_ | 965.1 ± 703.5 | 884.5 ± 642.6 | 0.057 |
| D_1mL_ | 880.1 ± 661.9 | 792.7 ± 584.5 | 0.067 |
| D_2mL_ | 833.3 ± 648.2 | 743.4 ± 556.7 | 0.090 |
| D_mean_ | 437.3 ± 400.6 | 401.1 ± 353.0 | 0.079 |

Abbreviations: D_mean,_ mean dose; D_max_, maximum dose
